# Case Report: Methanol poisoning mimicking acute coronary syndrome—a fatal case of massive intracranial hemorrhage on dual antiplatelet therapy

**DOI:** 10.3389/fcvm.2026.1801376

**Published:** 2026-04-15

**Authors:** Junping Zhu, Pan Wei, Yonghong Wang, Xianyi Yang

**Affiliations:** 1 Department of Emergency, Taihe Hospital Affiliated to Hubei University of Medicine, Shiyan, Hubei, China; 2 Hubei Provincial Clinical Research Center for Pneumoconiosis and Poisoning, Hubei Provincial Hospital of Integrated Chinese & Western Medicine, Wuhan, Hubei, China

**Keywords:** acute coronary syndrome, antiplatelet therapy, intracranial hemorrhage, methanol poisoning, misdiagnosis

## Abstract

This manuscript describes a fatal case of a 45-year-old man who presented with severe chest pain, hypertension (198/115 mmHg), and electrocardiogram changes mimicking acute coronary syndrome (ACS) 30 h after ingesting adulterated alcohol. Uniquely, chest pain was the sole initial manifestation, without visual disturbances, gastrointestinal symptoms, or any other classic methanol features, creating a clinically convincing ACS mimic. Despite negative coronary CT angiography, dual antiplatelet loading therapy (aspirin 300 mg and clopidogrel 300 mg) was administered based on clinical suspicion of ACS, together with low-molecular-weight heparin. Three hours later, the patient developed sudden unconsciousness with unequal pupils. Arterial blood gas analysis revealed severe high-anion-gap metabolic acidosis (pH 7.08, anion gap 34 mEq/L). Toxicology confirmed methanol poisoning (blood concentration 206.85 mg/dL). The diagnosis was prompted only after two coworkers presented simultaneously with visual symptoms and bilateral basal ganglia lesions on CT—a constellation not previously reported as the diagnostic trigger in ACS-mimicking methanol poisoning. Head CT showed subarachnoid hemorrhage, which rapidly progressed to massive right basal ganglia hemorrhage (9.0 × 3.3 cm) with intraventricular extension and surrounding hypodensity consistent with necrosis. Despite hemodialysis, ethanol infusion, and emergency craniotomy, the patient died 2 weeks later. Coagulation studies revealed acidosis-induced coagulopathy (PT 15.2 s, activated partial thromboplastin time 38.6 s, INR 1.3). Critically, what distinguishes this case from prior reports of methanol-associated basal ganglia hemorrhage is the synergistic pro-hemorrhagic state created by the combination of dual antiplatelet therapy (irreversible platelet inhibition), low-molecular-weight heparin (antithrombin III potentiation), and acidosis-induced coagulopathy, superimposed on methanol-induced endothelial injury—a fatal pharmacotoxicological interaction not previously described in a single case. Emergency physicians should consider toxicological etiologies in undifferentiated chest pain with metabolic derangements and obtain early arterial blood gas analysis to avoid such fatal diagnostic errors.

## 1 Introduction

Acute methanol poisoning is a rare but life-threatening toxicological emergency with high mortality if untreated (1). Methanol is metabolized via alcohol dehydrogenase to formaldehyde and subsequently to formic acid, the primary toxic metabolite responsible for severe metabolic acidosis and selective damage to the optic nerve and basal ganglia (2, 3). Classic presentations include visual disturbances, altered mental status, and gastrointestinal symptoms (4). However, atypical presentations may lead to diagnostic errors with devastating consequences. Although alcohol poisoning can cause abnormal electrocardiogram (ECG) findings (5–7), chest pain as the initial manifestation of acute methanol poisoning is exceedingly rare (8).

We report a case presenting primarily with severe chest pain and hypertension, initially misdiagnosed as acute coronary syndrome (ACS). The subsequent administration of dual antiplatelet therapy likely potentiated the fatal intracranial hemorrhage, a recognized complication of methanol toxicity. This case underscores the importance of early metabolic evaluation and cautious use of antithrombotic therapy when the diagnosis remains uncertain.

## 2 Case presentation

A 45-year-old previously healthy man presented to the emergency department (ED) with 6 h of severe, crushing substernal chest pain and profuse diaphoresis. He reported headache and dizziness but denied visual disturbances, nausea, or abdominal pain. His history revealed alcohol consumption at a social gathering approximately 30 h earlier, though the type and quantity were initially not disclosed. On examination, his blood pressure was 198/115 mmHg, heart rate 108 bpm, respiratory rate 22 breaths/min, and temperature 36.8 °C. He appeared diaphoretic and distressed. Neurological examination was unremarkable, with equal reactive pupils (3 mm bilaterally).

ECG showed sinus tachycardia with ST-segment depression (0.05–0.10 mV) in leads V4–V6 and T-wave flattening. Initial troponin I was elevated (1.1 ng/mL; reference <0.5), and creatine kinase-MB was borderline elevated (4.3 U/L; reference <4). Complete blood count revealed leukocytosis (13.2 × 10⁹/L). Initial coagulation parameters showed the following: PT 12.8 s (reference 11–13.5), activated partial thromboplastin time (aPTT) 28.4 s (reference 25.0–35.0), INR 1.1, and platelet count 185 × 10⁹/L (reference 150–400). Triple-rule-out CT angiography excluded coronary obstruction, aortic dissection, and pulmonary embolism. Echocardiography revealed lateral wall hypokinesis.

Unfortunately, arterial blood gas (ABG) analysis was not obtained at initial presentation due to the clinical focus on suspected ACS. Based on the clinical presentation suggesting non-ST-elevation myocardial infarction, dual antiplatelet therapy was administered (aspirin 300 mg and clopidogrel 300 mg loading doses), along with low-molecular-weight heparin and statin therapy. The cardiac catheterization laboratory was notified for emergent coronary angiography.

**Figure 1:**
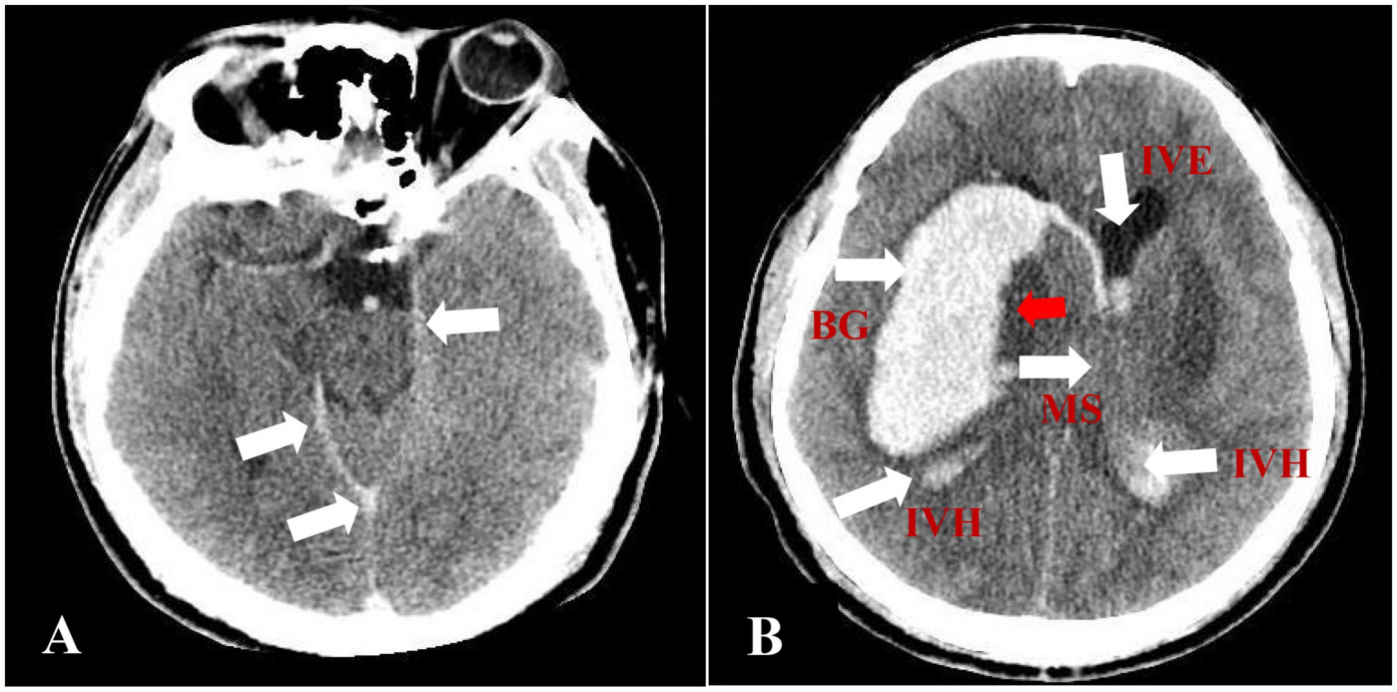
**(A)** Initial head CT (brain window settings: width 85 HU, level 35 HU) showing subarachnoid hemorrhage with high-density shadows along the tentorium cerebelli, longitudinal fissure, cerebral falx, and sulci (white arrows). **(B)** Follow-up CT 15 h later demonstrating massive right basal ganglia hemorrhage (9.0 × 3.3 cm) centered in the right putamen and caudate nucleus (labeled), with surrounding hypodensity consistent with necrotic change (arrowheads), intraventricular extension, and significant midline shift. The second image clearly demonstrates the classic methanol-associated hemorrhagic necrosis of the basal ganglia. Anatomical structures labeled: BG, basal ganglia (putamen and caudate nucleus); IVH, intraventricular hemorrhage; IVE, intraventricular extension; MS, midline shift.

Approximately 3 h after ED arrival, the patient suddenly became unresponsive with unequal pupils (left 4 mm, right 5 mm). Arterial blood gas analysis was performed at this time and revealed severe metabolic acidosis: pH 7.08, bicarbonate 8.5 mmol/L, base excess −18.5 mmol/L, anion gap 34 mEq/L, and lactate 5.8 mmol/L. Repeat troponin I showed elevation to 2.3 ng/mL. Emergent head CT demonstrated high-density shadows in the tentorium cerebelli, longitudinal fissure, cerebral falx, and some sulci, consistent with subarachnoid hemorrhage (Figure 1A).

Notably, two coworkers from the same gathering presented simultaneously to the ED with visual symptoms (blurred vision and decreased visual acuity) and bilateral basal ganglia hypodensities on CT, prompting strong suspicion of methanol poisoning. Toxicology confirmed blood methanol concentration of 206.85 mg/dL in our patient.

Treatment was initiated with sodium bicarbonate, ethanol infusion (10% solution), emergent hemodialysis, and folate/fomepizole. Repeat coagulation studies revealed mild prolongation—PT 15.2 s, aPTT 38.6 s, and INR 1.3, with stable platelet count of 178 × 10⁹/L—suggesting acidosis-induced coagulopathy. However, 15 h after admission, repeat head CT revealed a massive right basal ganglia hemorrhage (9.0 × 3.3 cm) with surrounding hypodensity consistent with necrosis, intraventricular extension, and midline shift (Figure 1B).

Emergency craniotomy was performed as a life-saving procedure despite the anticipated increased bleeding risk from combined antiplatelet therapy and coagulopathy. The operation confirmed massive hemorrhage with significant intraoperative bleeding (2,500 mL blood loss) and difficulty achieving hemostasis, consistent with synergistic pro-hemorrhagic effects. The patient remained comatose postoperatively and died 2 weeks later from multiorgan failure. Table 1 summarizes the complete timeline of diagnostic events and clinical course for this patient.

**Table 1:** Diagnostic timeline and clinical events.

Time	Event/finding
T −30 h	Alcohol consumption at social gathering
T 0 (ED arrival)	Chest pain, BP 198/115 mmHg, HR 108 bpm; ECG: ST depression V4V6; Troponin I 1.1 ng/mL; CK-MB 4.3 U/L; PT 12.8 s, aPTT 28.4 s, INR 1.1, PLT 185 × 10⁹/L
T + 30 min	CT angiography negative for coronary occlusion, aortic dissection, PE; Echo: lateral wall hypokinesis
T + 1 h	Dual antiplatelet therapy administered (ASA 300 mg + Clopidogrel 300 mg), LMWH, statin
T + 3 h	Sudden unconsciousness, unequal pupils; ABG: pH 7.08, HCO₃ 8.5 mmol/L, AG 34 mEq/L, lactate 5.8 mmol/L; Troponin I 2.3 ng/mL; head CT: subarachnoid hemorrhage; two coworkers present with visual symptoms
T + 4 h	Toxicology confirms methanol 206.85 mg/dL; PT 15.2 s, aPTT 38.6 s, INR 1.3, PLT 178 × 10⁹/L; treatment: bicarbonate, ethanol, hemodialysis, folate
T + 15 h	Repeat CT: massive right basal ganglia hemorrhage (9.0 × 3.3 cm) with necrosis, intraventricular extension, midline shift
T + 18 h	Emergency craniotomy performed; intraoperative blood loss 2,500 mL with difficulty achieving hemostasis
T + 2 weeks	Patient expired from multiorgan failure

ED, emergency department; BP, blood pressure; HR, heart rate; ECG, electrocardiogram; CK-MB, creatine kinase-MB; PT, prothrombin time; aPTT, activated partial thromboplastin time; INR, international normalized ratio; PLT, platelet count; PE, pulmonary embolism; ASA, aspirin; LMWH, low-molecular-weight heparin; ABG, arterial blood gas; HCO₃, bicarbonate; AG, anion gap.

## 3 Discussion

This case illustrates a critical diagnostic pitfall: methanol poisoning presenting as apparent ACS. The pathophysiology of chest pain in methanol toxicity is multifactorial and involves several mechanisms. Severe metabolic acidosis (pH < 7.10) causes direct myocardial depression and triggers compensatory sympathetic activation, leading to a catecholamine surge. This results in (1) increased myocardial oxygen demand from hypertension and tachycardia, (2) potential coronary vasospasm from catecholamine effects, and (3) type 2 myocardial injury from supply–demand mismatch (9). Together, these mechanisms explain the clinical presentation mimicking ACS in our patient, including severe chest pain, ST-segment changes, and troponin elevation (rising from 1.1 to 2.3 ng/mL), despite patent coronary arteries on CT angiography. Moreover, direct cardiac toxicity from formic acid may also contribute to myocardial dysfunction and the lateral wall hypokinesis observed on echocardiography (5).

Several factors contributed to the initial misdiagnosis: (1) classic ACS-like presentation in a middle-aged man; (2) supportive ECG findings and elevated cardiac biomarkers consistent with non-ST-elevation myocardial infarction; (3) absence of typical methanol symptoms, such as visual disturbance, at presentation; (4) incomplete exposure history—the patient did not initially volunteer information about alcohol consumption; and (5) critically, failure to obtain early arterial blood gas analysis. This last factor represents a crucial missed opportunity, as the severe high-anion-gap metabolic acidosis would have immediately prompted consideration of toxic alcohol ingestion.

What distinguishes our case from prior published reports of methanol-associated basal ganglia hemorrhage is the unique and fatal convergence of multiple pro-hemorrhagic factors resulting from a misdiagnosis-driven treatment error. Balodis et al. reported rapid deterioration with combined ischemic and hemorrhagic brain damage in methanol poisoning (10), while Safari et al. documented intracerebral hemorrhage in methanol-poisoned patients during the COVID-19 pandemic (11). Sohor et al. described spontaneous bilateral basal ganglia hemorrhage secondary to methanol poisoning (12). However, in none of these cases was antithrombotic therapy a contributing factor. In our patient, full-dose dual antiplatelet loading (aspirin 300 mg + clopidogrel 300 mg) combined with low-molecular-weight heparin was administered under the clinical impression of NSTEMI, creating an iatrogenic pro-hemorrhagic state superimposed on the intrinsic methanol-induced hemorrhagic diathesis. Furthermore, the diagnostic pathway in our case was uniquely unlocked by a coworker cluster presentation—two colleagues simultaneously presenting with classic methanol symptoms of visual disturbances and bilateral basal ganglia hypodensities—a scenario not described as a diagnostic trigger in any previously published ACS-mimicking methanol case. The massive intraoperative blood loss (2,500 mL) and inability to achieve hemostasis provided direct clinical evidence of synergistic coagulopathy, distinguishing the hemorrhagic course in our case from prior reports in the literature where antithrombotic therapy was not a factor.

Basal ganglia hemorrhage is a well-documented complication of methanol toxicity, occurring independent of antiplatelet therapy. Recent case reports have clearly demonstrated both ischemic and hemorrhagic basal ganglia involvement in methanol poisoning, with characteristic radiological patterns (10–13). The pathophysiology of methanol-induced intracranial hemorrhage involves endothelial damage from formic acid accumulation and acidosis-induced coagulopathy (11, 12). In our case, the administration of dual antiplatelet therapy before definitive diagnosis likely potentiated rather than caused the hemorrhagic progression. The combination of irreversible platelet inhibition from aspirin (COX-1 inhibition) and clopidogrel (P2Y12 receptor blockade), low-molecular-weight heparin (potentiating antithrombin III activity), and acidosis-induced coagulopathy (evidenced by prolonged PT 15.2 s, aPTT 38.6 s, INR 1.3) created a synergistic pro-hemorrhagic state (14). The massive intraoperative blood loss (2,500 mL) and difficulty achieving surgical hemostasis provide clinical evidence of this combined effect.

The CT findings in our patient were characteristic of methanol toxicity: bilateral putaminal hypodensities (necrosis) and hemorrhagic transformation of the basal ganglia. Brain CT in methanol poisoning typically shows symmetric low-density lesions in the putamen, caudate nucleus, and occasionally subcortical white matter, reflecting formate-mediated histotoxic hypoxia. Hemorrhagic transformation within these necrotic foci, as seen dramatically in our case, has been described in severe methanol poisoning and is associated with high mortality (15, 16).

MRI was not performed in our patient due to rapid clinical deterioration requiring emergency craniotomy. However, in patients stable enough to undergo MRI, characteristic findings would be expected: restricted diffusion [hypointense on apparent diffusion coefficient (ADC) map, hyperintense on diffusion-weighted imaging (DWI)] in the putamen and caudate nucleus, reflecting cytotoxic edema; T2/FLAIR hyperintensity throughout the affected basal ganglia; and gradient echo/susceptibility-weighted imaging hypointensity in areas of hemorrhagic necrosis (15–17). Of particular importance are the optic nerve findings. On DWI, the optic nerves may demonstrate restricted diffusion corresponding to axonal injury from formate toxicity, while T2-weighted sequences and gadolinium-enhanced sequences may show perineural enhancement and demyelination along the optic nerves and chiasm (16, 17). These optic nerve MRI findings, when present, are nearly pathognomonic of methanol poisoning and can help distinguish methanol toxicity from other causes of bilateral basal ganglia injury. The two coworkers in our case, who presented with visual symptoms and bilateral basal ganglia hypodensities on CT, would have been ideal candidates for MRI optic nerve evaluation; however, resource constraints precluded this. Future case reports should prioritize MRI acquisition, particularly DWI and ADC sequences of the optic nerves, in methanol-poisoned patients with visual symptoms who are clinically stable enough to undergo the examination (18).

The diagnosis in our case was ultimately prompted by the simultaneous presentation of two coworkers with classic methanol poisoning symptoms (visual disturbances and bilateral basal ganglia hypodensities on CT). This cluster presentation highlighted the importance of obtaining a detailed exposure history and considering environmental or occupational poisoning in undifferentiated presentations.

This case offers important lessons for emergency physicians managing undifferentiated chest pain. Toxicological etiologies should be considered in patients presenting with severe hypertension, disproportionate tachycardia, or unexplained metabolic derangements. The classic “too sick for the diagnosis” appearance should prompt broader differential diagnosis beyond the initial clinical impression. In particular, the combination of chest pain and severe metabolic disturbance warrants investigation for toxic ingestions alongside traditional cardiac etiologies.

Early arterial blood gas analysis is essential in critically ill patients presenting with chest pain, particularly when the clinical picture appears disproportionate to initial findings. High-anion-gap metabolic acidosis should immediately trigger consideration of toxic alcohol ingestion (methanol, ethylene glycol), diabetic ketoacidosis, lactic acidosis, or other metabolic emergencies. In our case, ABG was not obtained until 3 h after arrival, by which time dual antiplatelet therapy had already been administered and the diagnostic window had narrowed considerably. Routine early metabolic screening in atypical or severe presentations might have prevented this catastrophic outcome.

Caution should be exercised with empirical dual antiplatelet therapy when diagnosis remains uncertain, particularly in patients with neurological symptoms, unexplained metabolic abnormalities, or atypical presentations. While timely antiplatelet therapy is life-saving in true ACS, premature administration in alternative diagnoses can have catastrophic consequences, as demonstrated in this case. The irreversible nature of platelet inhibition from aspirin and clopidogrel means that once administered, the pro-hemorrhagic effects cannot be rapidly reversed. A brief period of diagnostic evaluation—including basic metabolic assessment—may prevent such iatrogenic complications without significantly compromising outcomes in confirmed ACS cases.

Thorough exposure history remains a cornerstone of emergency medicine practice. This should routinely include recent alcohol consumption, occupational exposures, and inquiry about similar symptoms in contacts or coworkers. Our patient did not initially disclose alcohol consumption, and the diagnosis was only established when coworkers presented with classic symptoms. Active questioning about potential exposures—particularly in cases that do not fit typical patterns—may reveal critical diagnostic information that patients do not spontaneously volunteer.

This case report has several limitations. MRI was not performed due to the patient's critical condition, rapid deterioration, and need for emergency intervention, precluding detailed characterization of the basal ganglia pathology and optic nerve injury. DWI, ADC mapping, and T2-weighted sequences would have provided valuable insights. In particular, DWI and ADC sequences of the optic nerves—potentially revealing restricted diffusion corresponding to formate-induced axonal injury—and gadolinium-enhanced sequences showing perineural enhancement could have provided important pathophysiological insights and pathognomonic evidence of methanol toxicity. Serial methanol levels were not obtained to document clearance kinetics. The two coworkers did not undergo MRI due to resource constraints and were treated with conservative management; their imaging contribution is therefore limited to CT findings.

## 4 Conclusion

Methanol poisoning can present atypically with severe chest pain mimicking ACS. Basal ganglia hemorrhage is a recognized complication of methanol toxicity that may be potentiated by antiplatelet therapy. This tragic case demonstrates that premature dual antiplatelet and antithrombotic therapy in unconfirmed ACS can have fatal consequences when the underlying etiology predisposes to hemorrhage. What makes this case particularly instructive is the unique convergence of methanol-induced hemorrhagic necrosis, full antithrombotic loading given under a misdiagnosis, and a diagnostic breakthrough triggered by a coworker cluster presentation—a combination not previously described in the literature. Early arterial blood gas analysis, comprehensive history-taking including exposure assessment, and diagnostic humility are essential to avoid such catastrophic errors in emergency medicine. When faced with atypical presentations of suspected ACS, particularly with severe metabolic derangements, emergency physicians should consider alternative diagnoses before committing to irreversible antiplatelet therapy.

## Data Availability

The original contributions presented in the study are included in the article/supplementary material, further inquiries can be directed to the corresponding author.
